# Drug Regulatory-Compliant Validation of a qPCR Assay for Bioanalysis Studies of a Cell Therapy Product with a Special Focus on Matrix Interferences in a Wide Range of Organ Tissues

**DOI:** 10.3390/cells12131788

**Published:** 2023-07-05

**Authors:** Hannes M. Schröder, Elke Niebergall-Roth, Alexandra Norrick, Jasmina Esterlechner, Christoph Ganss, Markus H. Frank, Mark A. Kluth

**Affiliations:** 1 RHEACELL GmbH & Co. KG, 69120 Heidelberg, Germany; 2 Department of Dermatology, Brigham and Women’s Hospital, Harvard Medical School, Boston, MA 02115, USA; 3 Harvard Stem Cell Institute, Harvard University, Cambridge, MA 02138, USA; 4 Transplant Research Program, Boston Children’s Hospital, Harvard Medical School, Boston, MA 02115, USA; 5 School of Medical and Health Sciences, Edith Cowan University, Perth, WA 6027, Australia

**Keywords:** ABCB5, biodistribution, cell therapy, HSSATA17 sequence, mesenchymal stromal cells, quantitative polymerase chain reaction, spike recovery, tissue matrix effects

## Abstract

Quantitative polymerase chain reaction (qPCR) has emerged as an important bioanalytical method for assessing the pharmacokinetics of human-cell-based medicinal products after xenotransplantation into immunodeficient mice. A particular challenge in bioanalytical qPCR studies is that the different tissues of the host organism can affect amplification efficiency and amplicon detection to varying degrees, and ignoring these matrix effects can easily cause a significant underestimation of the true number of target cells in a sample. Here, we describe the development and drug regulatory-compliant validation of a TaqMan^®^ qPCR assay for the quantification of mesenchymal stromal cells in the range of 125 to 20,000 cells/200 µL lysate via the amplification of a human-specific, highly repetitive α-satellite DNA sequence of the chromosome 17 centromere region HSSATA17. An assessment of matrix effects in 14 different mouse tissues and blood revealed a wide range of spike recovery rates across the different tissue types, from 11 to 174%. Based on these observations, we propose performing systematic spike-and-recovery experiments during assay validation and correcting for the effects of the different tissue matrices on cell quantification in subsequent bioanalytical studies by multiplying the back-calculated cell number by tissue-specific factors derived from the inverse of the validated percent recovery rate.

## 1. Introduction

The preclinical xenotransplantation of human cells into immunodeficient mice is an essential component of human-cell-based therapy development [1,2,3]. By determining the distribution and retention of cell products after administration to mice, important questions can be addressed, such as whether the delivered cells actually reach the target site(s), whether they engraft in sufficient numbers to produce the desired effect, whether they are distributed to unwanted non-target tissues, and how long they persist in the host organism. Answering these questions is critical to uncovering the underlying mechanism(s) of action, exploring interventions to improve cell engraftment ratios, and evaluating the biosafety profiles of human cell-based therapy strategies, as required by the regulatory authorities prior to any in-human use [3,4,5].

Bioanalysis studies evaluating the distribution, persistence, and clearance of cell therapy products place special demands on the analytical method. First, the method must be highly specific and sensitive to detect and quantify very small populations of human cells that are vastly outnumbered by the mouse cells surrounding them. Secondly, because transplanted cells can be distributed in an inhomogeneous manner within organs [6,7], the method must ensure that its accuracy is not biased as a result of sampling errors due to a non-random cell distribution. Thirdly, the method should ideally not require the cells to be pre-labeled, since this may affect their viability and/or functionality [8,9,10] and would therefore require the comparability of the labeled cells to the (unlabeled) cells intended for human use to be established [10,11,12]. Given these requirements, quantitative polymerase chain reaction (qPCR) has evolved as a method of choice in the bioanalysis of cell therapy products [13,14,15]. qPCR assays enable specific and extremely sensitive tracking and absolute quantification of the donor cells via the detection of species-specific DNA sequences within the whole host organ. This makes qPCR suitable for the rapid and convenient systematic quantification of unlabeled donor cells even at very low cell numbers in a broad range of host organs and tissues [4,5,16,17].

However, the performance of a qPCR assay depends on a number of parameters, including but not limited to tissue sampling and nucleic acid extraction methods, choice of primers and probes, selection of reagents and reaction conditions, determination of the quantification cycle (Cq) values, and matrix effects [18,19]. Therefore, any qPCR method must be validated to ensure the reliability of the data that will be obtained. Remarkably, however, despite the growing number of qPCR applications in regulated bioanalysis, and even though qPCR is one of the officially recognized methods for determining the pharmacokinetics of cell therapy products [11], regulatory guidance on the use of qPCR is limited [13,20,21,22,23]. The International Council for Harmonisation of Technical Requirements for Pharmaceuticals for Human Use (ICH) Guideline M10 on Bioanalytical Method Validation and Study Sample Analysis [24,25], which recently superseded the guidelines on bioanalytical method validation released by the European Medicines Agency (EMA) [26] and the US Food and Drug Administration (FDA) [27], focuses on pharmacokinetic methods that are suitable for classical small-molecule drugs and large-molecule biologics, such as chromatographic methods and ligand-binding assays, but do not address the specific requirements for the proper validation of PCR assays [13,20,21].

Another challenge in bioanalytical qPCR studies is that the different organs and tissues of the host organism can affect amplification efficiency and amplicon detection to varying degrees, and ignoring such matrix effects can easily lead to an underestimation of the true number of target cells in a sample. Matrix effects have been studied predominantly in environmental microbiology, microbial food safety and forensic analyses, where the amounts of target nucleic acids are often extremely small and the matrices are particularly diverse and challenging [28,29,30]. In contrast, with the exception of forensically and microbiologically relevant body fluids and secretions, as well as food safety-relevant matrices such as muscle tissue and milk, the effects of mammalian matrices on qPCR results have been assessed and discussed only in a very limited manner and only for a limited number of tissue types [14,16,31,32,33,34].

With particular attention to these challenges, here, we describe the development and validation of a qPCR assay for the reliable detection and quantification of human cells in mouse tissues and blood. The validation included a systematic assessment of the matrix effects of 14 different mouse tissues, including blood. This assay enabled the generation of preclinical biodistribution data acceptable to regulatory authorities [35], which were required for the approval of a medicinal product based on skin-derived ABCB5^+^ mesenchymal stromal cells (MSCs) [36,37] to be tested in clinical trials. The insights presented here may also be relevant for a range of other scientific contexts and purposes.

## 2. Materials and Methods

### 2.1. Assay Design

A TaqMan^®^ qPCR assay was developed for the detection and quantification of human ABCB5^+^ MSCs in mouse tissues via the detection of a DNA sequence of human α-satellite DNA [38]. As an internal control of efficient DNA extraction from mouse tissues, in mouse tissue homogenates, a mouse-specific DNA sequence of the prostaglandin E receptor 2 (*PTGER2*) gene [39] was also detected. The assays were run in a certified GLP-compliant test facility (Accelero Bioanalytics, Berlin, Germany). Reporting follows the MIQE (Minimum Information for Publication of Quantitative Real-Time PCR Experiments) Guidelines where applicable [40].

### 2.2. Primers and Probes

Human-specific DNA was detected via the amplification of a sequence of the α-satellite DNA on chromosome 17 (HSSATA17, GenBank Acc. No. M13882), using the forward primer GGGATAATTTCAGCTGACTAAACAG, reverse primer AAACGTCCACTTGCAGATTCTA, and 6-carboxyfluorescein-labeled probe CACGTTTGAAACACTCTTTTTGCA carrying the Black Hole Quencher^®^ BHQ^®^-1. Mouse-specific DNA was detected via the amplification of a mouse-specific DNA fragment of the *PTGER2* gene using the forward primer TACCTGCAGCTGTACGCCAC, reverse primer GCCAGGAGAATGAGGTGGTC, and carboxytetramethylrhodamine-labeled probe CCTGCTGCTTATCGTGGCTG carrying BHQ^®^-2. The specificity of these sequences was confirmed previously [38,39]. All primers and probes were supplied by Microsynth (Balgach, Switzerland). Primer and probe lyophilizates were dissolved in DNase-free water to prepare 100 µM stock solutions, from which 18 µM (primers) and 5 µM (probes) working solutions were prepared.

### 2.3. Human ABCB5^+^ MSCs

Human ABCB5^+^ MSCs were derived from skin samples taken from human subjects aged ≤ 50 years undergoing abdominoplasties or other plastic surgeries providing leftover skin tissue. Skin sampling was performed in accordance with the German Act on Organ and Tissue Donation, Removal and Transplantation after written informed consent was obtained from each donor. Skin processing and cell production were carried out in an EU-GMP grade A cabinet in a grade B clean room facility under laminar air flow according to a validated GMP-compliant manufacturing protocol, as described previously [36]. In brief, skin tissue was freed from excess subcutaneous tissue, disinfected, washed, dissected into equal pieces (approximately 2.5 cm^2^), and enzymatically (collagenase followed by animal component-free trypsin) digested. After the filtration and washing/centrifugation of the filtrates, the cells were expanded as unsegregated cultures in monolayer culture via serial passaging upon adherence selection in an in-house MSC-favoring culture medium (Ham’s F-10 supplemented with fetal calf serum, L-glutamine, fibroblast growth factor 2, HEPES, hydrocortisone, insulin, glucose, and phorbol myristate acetate) at 3.1% CO_2_, 90% humidity, and 37 °C for up to 16 passages, provided that no changes in cell morphology or growth behavior had occurred. From the primary cultures, ABCB5^+^ MSCs were isolated via antibody-coupled magnetic bead sorting using a mouse anti-human monoclonal antibody directed against the extracellular loop 3 of the ABCB5 molecule [41] (Maine Biotechnology Services, Portland, Maine; GMP-compliant purification: Bibitec, Bielefeld, Germany). After the enzymatic detachment of the beads from the cell surface, the isolated ABCB5^+^ MSCs were cryo-preserved in CryoStor^®^ CS10 freeze medium (BioLife Solution, Bothell, WA, USA) containing 10% dimethyl sulfoxide and stored in the vapor phase of liquid nitrogen. Batch homogeneity, biological functionality (potency), and product safety were monitored and ensured using mandatory in-process controls in each production step and release tests following GMP-compliant procedures with validated, predefined acceptance criteria, as detailed previously [36].

For spiking, ABCB5^+^ MSC suspensions were thawed at 37 °C in a thermal mixer. To remove the cell debris and free, degraded DNA, the cell suspension was washed with 1× PBS. After centrifugation for 5 min at 500× *g*, the cell pellet was taken up in PBS to adjust the cell concentration to approximately 2000 cells/µL. The effective cell concentration, as assessed through cell counting under a light microscope using a hemocytometer, was 2008 cells/µL. The cell suspension was aliquoted and stored at −80 to −60 °C.

### 2.4. Mouse Tissue Sampling

Animal breeding, care, necropsy, and tissue collection were conducted by a specialized contract research organization (Preclinics, Potsdam, Germany). SCID/beige mice (21–23 weeks old, 5 male, 5 female) were anesthetized with isoflurane, and whole blood was collected via cardiac puncture into EDTA collection tubes. The animals were then sacrificed with an overdose of xylazine and the following tissues were collected: mouse skin (neck region), thigh muscle (M. quadriceps femoris), lymph nodes (cervical, axial, and inguinal lymph nodes, pooled), liver, spleen, lung, brain, bone (femur) including marrow, kidneys, thymus, thyroid, and ovaries/testes.

To avoid contamination with human DNA and cross-contamination between animals, necropsy and tissue collection were performed under a laminar airflow workbench. All work areas were disinfected before work commenced. The personnel wore disposable lab coats, hoods and face masks, and two pairs of gloves. The gloves were disinfected before work commenced and the outer pair of gloves were changed after each animal. A separate autoclaved dissection set was used for every four animals (one cage) and was disinfected after each animal. The tissue pads for necropsy were changed after each animal. The tissues were collected in DNAse-free collection tubes.

### 2.5. Preparation of Standards

Standards were prepared and the samples were lysed and DNA extracted using the NucleoSpin^®^ 96 Tissue kit (Macherey-Nagel, Düren, Germany) according to the manufacturer’s instructions, following the protocols described below.

#### 2.5.1. Calibration Standards and Quality Control Standards

Seven calibration standards (range: 125 to 20,000 ABCB5^+^ MSCs) and five quality control standards (range: 125 to 15,000 ABCB5^+^ MSCs) were prepared through the serial dilution of ABCB5^+^ MSC suspension in Tris-EDTA buffer. Tris-EDTA buffer without cells was used as blank samples. In total, 20 microliters of each cell suspension or blank sample was added to 180 µL lysis buffer T1, followed by the addition of 25 µL proteinase K. The samples were lysed at 56 °C for 15–30 min. The lysates were stored at −80 to −60 °C until DNA was eluted using 60 µL of pre-heated (70 °C) elution buffer BE.

#### 2.5.2. Tissue Quality Control Standards

Mouse tissues were taken up in lysis buffer T1 (amount as required to adjust the intended tissue concentration, see Table S1) into homogenization tubes filled with ceramic beads (for soft tissues) or steel beads (for bone tissue, with the addition of lysis buffer T1 only after two “dry” homogenization cycles without lysis buffer) and homogenized in a Precellys Evolution homogenizer (Bertin Technologies, Frankfurt, Germany) at 6000 rpm and room temperature for 20 s per cycle with at least a 30 s pause between cycles. If more than two cycles were required for complete homogenization (see Table S1 for the total number of cycles for the different tissues), the samples were cooled between each cycle. The homogenates (225 µL each) were spiked with 25 µL cell suspension containing 0, 125, 625, or 5000 ABCB5^+^ MSCs. The spiked homogenates were homogenized for a further cycle, and then 25 µL proteinase K was added. The samples were lysed at 56 °C for 15–30 min.

For the preparation of the blood quality control standards, 100 µL EDTA blood was spiked with 20 µL cell suspension (containing 0, 125, 625, or 5000 ABCB5^+^ MSCs), filled up to 400 µL with PBS, and then 25 µL (assays 1 and 2) or 50 µL (assay 3) proteinase K and 400 µL binding buffer BQ1 was added. The samples were lysed at room temperature for 5 min (assays 1 and 2) or 30 min (assay 3) followed by 70 °C for 15 min.

All lysates were stored at −80 to −60 °C until the DNA was eluted using 60 µL of pre-heated (70 °C) elution buffer BE.

All steps related to tissue transfer, cutting, splitting, lysis, DNA extraction, and transfer of the eluate to the qPCR plate were performed under a laminar airflow workbench using sterile, disposable equipment. The personnel wore two pairs of sterile gloves; the outer pair and the tools used to split and transfer the samples were changed between each sample. Where tissue samples had to be sectioned, sterile, DNA-free, 24-well culture plates were used as a sectioning surface.

#### 2.5.3. Freeze–Thaw Stability

To test for the freeze–thaw stability of the extracted DNA, DNA eluted from the quality control standards was divided into two aliquots. One set of aliquots was stored at 2 to 8 °C and the other set at −25 to −15 °C for at least 12 h prior to analysis.

### 2.6. Amplification

The master mix for a singleplex PCR reaction consisted of 5 µL GoTaq Probe qPCR Master Mix (Promega, Madison, WI, USA) each supplemented with 0.5 µL of DNase-free water, 18 µM forward primer, 18 µM reverse primer, and 5 µM probe. The master mix (7 µL) was mixed with 3 µL of a 10-fold dilution (in DNase-free water) of template DNA, resulting in a reaction volume of 10 µL. For the no-template controls, DNAse-free water was used. The amplifications were run on an Applied Biosystems^TM^ ViiA^TM^ 7 Dx Real-Time PCR instrument (Thermo Fisher Scientific, Langenselbold, Germany). The cycling program consisted of an initial denaturation step of 10 min at 95 °C followed by 45 cycles of 15 s at 95 °C and 1 min at 60 °C. For the detection of human DNA, the samples were assayed in triplicate, except for the samples spiked with lower cell numbers (250 and below), which were assayed in sextuplicate. For the detection of mouse DNA, the samples were assayed in monoplicate.

### 2.7. Assay Validation and Acceptance Criteria

The assay was validated in accordance with the general requirements for bioanalytical method validation set out in the EMA Guideline on Bioanalytical Method Validation [26], recently superseded by the ICH Guideline M10 on Bioanalytical Method Validation and Study Sample Analysis [24], evaluating the parameters linearity, intra-assay and inter-assay accuracy and precision, specificity, freeze–thaw stability, and tissue matrix effects against predefined acceptance criteria (Table 1).

#### 2.7.1. Linearity

Calibration curves were generated by plotting the Cq number against the logarithm of the cell numbers in the calibration standards. A linear calibration function,

Cq = slope(log(cells)) + y-intercept(1)

was fitted via least-squares regression. Linearity was assumed if the correlation coefficient r^2^ was ≥0.95. The equation of the best-fit line was used to back-calculate the numbers of cell equivalents. Assay efficiency (E) was calculated as:

E = (10^(−1/slope)^ − 1) × 100. (2)

#### 2.7.2. Accuracy and Precision

Intra-assay and inter-assay accuracy (expressed as percent bias of the calculated cell number from the nominal cell number; acceptance criterion: within ±40%) and precision (expressed as the percent coefficient of variation (CV) between a series of measurements of the same sample; acceptance criterion: ≤40%) were determined based on the calibration standards and quality control standards assayed in triplicate or sextuplicate (as specified above). Three independent runs were performed on three different days. For assay validation, at least 75% of the calibration standard samples and at least 67% of the quality control standards had to meet the acceptance criteria for accuracy and precision.

#### 2.7.3. Specificity

To demonstrate specificity of the assay, the no-template controls were required to give either no amplification signal or a Cq value unequivocally distinguishable from the lower limit of quantification (LLOQ). To confirm that the assay specifically quantifies human cells even in the presence of mouse cells, tissue blanks, i.e., mouse tissue samples not spiked with human cells, were also analyzed.

#### 2.7.4. Tissue Matrix Effects

The effects of the mouse tissues on the DNA extraction and assay performance were assessed by determining the spike recovery in the tissue quality control standards (mouse tissues spiked with ABCB5^+^ MSCs). Three independent runs were performed on three different days. Spike recovery rates (ratio of the measured to the nominal cell count, expressed as a percentage of the nominal cell count) were used to calculate matrix factors (defined as the reciprocal of the percent recovery rate) for each tissue analyzed.

## 3. Results

### 3.1. Linearity and Quantification Range

The linear regression parameters of the calibration curves of the three validation assays (assays 1–3; Table 2) disclose a linear correlation between the log cell number and the Cq value (correlation coefficient r^2^ = 0.990, 0.971, and 0.992 for the three validation assays) over the quantification range from 125 human MSCs/200 µL lysate (=LLOQ) to 20.000 human MSCs/200 µL lysate (= upper limit of quantification, ULOQ).

### 3.2. Accuracy and Precision

Accuracy and precision were assessed in three independent validation assays based on the calibration standards and quality control standards run on three different days.

Of the 81 calibration standard replicates measured, all yielded signals. Four values (each two of six replicates of Cal 7 with a nominal cell number =125 in both assays 1 and 2) were excluded from the calculation to improve curve fitting. In assay 2, two replicates of Cal 6 (nominal cell number: 250) showed a bias >40% (back-calculated cell numbers: 490 and 448) but were still included in the calculations. This resulted in a high intra-assay CV of 41%, which just missed the range of acceptance. Overall, the intra-assay accuracy ranged from −21% to 25% bias and the intra-assay precision from 2% to 41% CV. The inter-assay accuracy ranged from −10% to 13% bias and the inter-assay precision from 9% to 22% CV, with all values within the range of acceptance (Table 3).

Of the 54 quality control standard replicates measured, all yielded signals. Six values (all three replicates of QC 3 with a nominal cell number =1250 in assay 1, two replicates of QC 4 with a nominal cell number = 650 in assay 1, and one replicate of QC 1 with a nominal cell number =15,000 in assay 2) were excluded because of the high bias of the back-calculated value from the nominal value. Overall, the intra-assay accuracy ranged from −36% to 36% bias and the intra-assay precision from 5% to 36% CV, with all values within the range of acceptance. The inter-assay accuracy ranged from −18% to 8% bias and the inter-assay precision from 11% to 27% CV, with all values also within the range of acceptance (Table 4).

### 3.3. Specificity

The no-template controls either gave no amplification signal (Cq value > 40, assay 3) or their mean Cq value was unequivocally distinguishable from that of the LLOQ (QC 5; assays 1 and 2) (Table 4). Of the 251 tissue blank replicates measured in total, 98 (39%) gave no amplification signal. The other replicates gave weak signals, with mean back-calculated cell counts ranging from 0 to 10 cells (corresponding to 0–8% of the LLOQ) in nearly all the tissues, except for the liver (21 cells, 17% of the LLOQ) and muscle (22 cells, 18% of the LLOQ) (Table S2).

### 3.4. Freeze–Thaw Stability of Extracted DNA

The freeze/thaw stability of the extracted DNA for one freeze/thaw cycle was assessed in quality control standard lysates stored at −25 to −15 °C. The bias of the cell number measured in these aliquots from the cell number measured in the aliquots that were stored at 2 to 8 °C ranged between −14% and 14% (Table S3).

### 3.5. Tissue Matrix Effects

**Figure 1:**
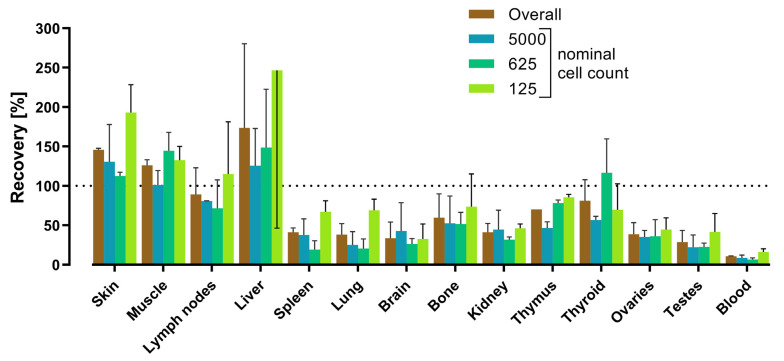
Spike recovery rates for various mouse tissues and blood spiked with 5000, 625, 125, and 0 (blank samples) human skin-derived ABCB5^+^ MSCs per 200 µL lysate, shown for the overall quantification range and each nominal cell count. Data are means with SD from two independent assays (assays 2 and 3; see Table 5) run on two different days. Due to space limitations, the SD bar for liver, 125 cells, is displayed in a downward direction. Within each assay, samples spiked with 5000 and 625 cells were assayed in triplicate, and samples spiked with 125 cells and blank samples (not shown) in sextuplicate.

Tissue matrix effects were assessed in three independent assays (assays 2–4) of the tissue quality control standards run on three different days (Table S2). Since in assay 4 the spike recovery rates for almost all the tissues were considerably lower as compared to those in assays 2 and 3 (Table 5), inefficient DNA extraction was assumed for assay 4. Therefore, the data were re-analyzed for assays 2 and 3 alone (Table 5). Spike recovery varied between the different tissue types, with the mean recovery rates (assays 2 and 3) ranging from 11% (blood) to 174% (liver) (Table 5). For most of the tissues (i.e., all except for the muscle, brain, and thyroid), the spike recovery rate was highest in the samples spiked with the lowest cell numbers (nominal cell count = 125 cells/200 µL tissue lysate) (Figure 1).

In an attempt to improve spike recovery from the blood samples, modified sample preparation and DNA extraction protocols were tested (Table S4). However, neither increasing the amount of proteinase K and extending the incubation time at room temperature (assay 4) nor various modifications, such as reducing the amount of blood, PBS, binding buffer BQ1, and/or the time of incubation at room temperature, resulted in higher recovery rates but rather tended to further decrease the spike recovery.

## 4. Discussion

Although qPCR-based assays have emerged as an important bioanalytical method for assessing the pharmacokinetics of human-cell-based medicinal products [4,5,16,21], the regulatory guidelines on the validation of bioanalytical methods released by the EMA, FDA, and ICH [24,25,26,27] focus on methods suitable for small- and large-molecule drugs such as chromatographic and ligand-binding assays. While the basic concepts and parameters of method validation described in these guidelines can be adapted to cell quantification via qPCR, in the absence of specific regulatory recommendations including definitive acceptance criteria for a validated qPCR assay, researchers must rely on published evidence from bioanalytical scientists, as well as recently issued best practice recommendations [15,20,42] and white papers from scientific networks [21,22,23,43].

The validation presented here followed the validation parameters set out in the EMA Guideline on Bioanalytical Method Validation [26], recently superseded by the ICH Guideline M10 on Bioanalytical Method Validation and Study Sample Analysis [24], using predefined acceptance criteria (Table 1). The acceptance criteria for accuracy and precision, which were set to within ±40% and ≤40%, respectively, are within the range of those recently recommended by the European Bioanalysis Forum [22]. The data obtained show that human MSCs can be detected and quantified with acceptable linearity, accuracy, and precision within the range of 125 (LLOQ) to 20,000 (ULOQ) cells/200 µL lysate (Table 1).

When developing and validating a bioanalytical assay, it is essential to match the setup to the actual study in which the assay will be used [24,25]. In addition to factors such as the intended mouse strain(s) or the amount of available sample material, an important aspect of bioanalytical cell detection via qPCR is the fact that many components in biological matrices can bias the cell quantification results. Such components include molecules that can impair DNA extraction or, after being co-extracted with the DNA, can affect amplification through disturbing annealing of the primers or inhibiting the DNA polymerase or interfere with amplicon detection via quenching fluorescence or interaction with the fluorophore [16,19,30]. In contrast to environmental, food, or forensic qPCR, there is only limited information on substances that may interfere with qPCR-based cell detection in the field of bioanalysis. The known inhibitory molecules present in bioanalytically relevant tissues and body fluids include molecules present in the skin, muscle, and bone such as melanin, myoglobin, collagen, and calcium ions, as well as various blood constituents, including added anticoagulants (Table 6). However, the issue of matrix effects on cell quantification via qPCR must be considered for all tissue types of the body. The yield and quality of extracted genomic DNA can vary widely depending on the physical and biochemical nature of each tissue [16,31], and the tissue from which the DNA was extracted can have a significant effect on the efficiency, accuracy, and precision of the qPCR assay [34]. Therefore, current regulatory guidelines [24,25] and best practice recommendations [15,20,21,22,43] advise researchers to assess potential matrix effects by determining the recovery of the target DNA spiked into each tissue of interest during assay validation, whereby recovery rates in a wide range between 30% and 100% are considered to be expected [15,20].

Our experiments conducted on a wide variety of tissues revealed an even wider range of recovery rates between the various tissue types, with 2 out of the 14 tissues showing recovery rates below 30% (1 of which, the testes, had a recovery rate of 29%, just missing the range) and 3 out of the 14 tissues showing recovery rates above 100% (Figure 1). The lowest recovery was achieved in the blood samples, in which only 11% of the spiked cells were detected. In this respect, it is important to note that blood is generally considered to be a particularly challenging matrix [49,52]. PCR mixtures based on *Taq* DNA polymerases have been reported to be inhibited in the presence of 1% [53] or even 0.004% (*v*/*v*) EDTA whole blood [54], and whole blood components co-extracted with the DNA can cause several negative effects such as a loss of the amplifiable target DNA, a reduction in amplification efficiency, and the quenching of fluorescence [48,49,50,51]. On the other hand, a thorough purification of DNA extracts can result in significant loss of DNA, which can also reduce recovery rates to as low as 10% [30]. Modifications to the extraction protocol, such as increasing the volume of proteinase K and the incubation time, have been reported to increase the yield of amplifiable DNA from blood samples [55] but did not improve the recovery rates of human ABCB5^+^ MSCs spiked into mouse blood samples in the present study (Table S4).

Overall, the data from the spike-and-recovery experiments demonstrate that a qPCR assay for bioanalytical studies runs the risk of substantially underestimating or even overestimating the cell numbers if the potential matrix effects due to physical and biochemical differences between the various tissues of the host organisms are not taken into account. Interestingly, a trend towards higher recovery rates at lower spiked cell concentrations was observed in almost all the tissues in our assay (Figure 1). Although we have no causal explanation for this observation, this trend suggests that the risk of underestimation decreases towards the lower limit of the validated quantification range.

In any case, while regulatory authorities require the determination of tissue matrix effects as part of bioanalytical method validation [24,27], they do not provide guidance on how to handle the results. An ideal, thorough assay optimization to minimize the impact of the different tissue matrices on cell quantification results would require an elaborate, costly, and animal-intensive program to evaluate the amount, integrity, and purity of the DNA extracted in different ways and/or to assess the amplification efficiency for, e.g., different buffer compositions and/or added facilitators [19,30]. Such complex programs, which would have to be performed and validated separately for each tissue of interest, would be beyond the resources of a research group or cell-therapy-developing company, especially as bioanalytical studies require the analysis of a wide range of different tissues. Instead, for reasons of feasibility, in the sense of a fit-for-purpose approach [24,25], we suggest correcting for the effects of the different tissue matrices on cell quantification in the subsequent actual sample measurements by multiplying the back-calculated cell count by a matrix-specific factor, representing the reciprocal of the percent recovery rate (Table 5).

An important question in bioanalytical studies is whether the cells that are detected are actually alive and, as such, potentially active, or whether they are not [56]. Researchers need to note that, unlike other cell detection methods such as flow cytometry and optical imaging [4,57], qPCR cannot distinguish between nucleic acids extracted from live cells, cell fragments, or cell corpses engulfed by local macrophages [5]. However, in vivo, genomic DNA is rapidly degraded upon cell death as an intrinsic part of the apoptotic program and/or by the lysosomal DNAses of cells that have phagocytosed the apoptotic or necrotic cell corpses [58,59]. Therefore, it is widely accepted that the inadvertent quantification of DNA isolated from dead cells is rather unlikely and, in the context of human cell xenotransplantation, the presence of dead human cells or residual human genomic DNA would not be expected to significantly bias the quantification of live human cells [14,56,60,61]. As with the distinction between living and dead cells, qPCR is also not able to distinguish between proliferating and non-proliferating cells. For cells that are intended for use as cell therapy products, however, any potential for unwanted proliferation must be excluded [5,62]. Therefore, in bioanalytical studies assessing the biosafety of a cell-based therapy, cell quantification via qPCR needs to be complemented with an appropriate method to assess the proliferative activity of the detected cells, e.g., the immunohistochemical double staining of tissue slides with a human-specific antibody and an antibody against a proliferation marker such as Ki67 [35].

## 5. Conclusions

From the perspective of cell therapy development, the data presented demonstrate that the efficacy and safety of stromal cell therapies in xenotransplantation models must be evaluated on a tissue-specific basis. The biodistribution and dose–response relationships for human cell-based medicinal products obtained in animal models using a validated qPCR assay must be considered in a differentiated manner due to different tissue-specific matrix interferences, which can affect cell recovery rates to very different extents. By contrasting the results from the different tissues, the present study suggests the use of tissue-specific matrix factors to correct for the effects of the different tissue matrices on cell quantification in the subsequent actual sample measurements.

## Figures and Tables

**Table 1:** Assay validation.

Parameter	Acceptance Criteria	Results
Linearity	r^2^ ≥ 0.95	0.971–0.992
Accuracy	Bias of the calculated cell number from the nominal cell number within ±40%	Cals: intra-assay: −21%–25% inter-assay: 10%–13% QCs: intra-assay: −36%–36% inter-assay: −18%–8%
	≥75% of Cals and ≥67% of QCs meet the acceptance criterion for inter-assay accuracy	100% of Cals and 100% of QCs met the acceptance criterion for inter-assay accuracy
Precision	CV between a series of measurements ≤40%	Cals: intra-assay: −21%–25% inter-assay: 10%–13% QCs: intra-assay: −36%–36% inter-assay: −18%–8%
	≥75% of Cals and ≥67% of QCs meet the acceptance criterion for inter-assay precision	100% of Cals and 100% of QCs met the acceptance criterion for inter-assay precision
Quantification range	LLOQ = lowest cell concentration quantified with acceptable accuracy and precision	LLOQ: 125 human MSCs in 200 µL lysate
	ULOQ = highest cell concentration quantified with acceptable accuracy and precision	ULOQ: 20,000 human MSCs in 200 µL lysate
Specificity	No-template controls give either no amplification signal or a Cq value unequivocally distinguishable from the LLOQ	Assays 1 and 2: Cq value unequivocally distinguishable from the LLOQ Assay 3: No amplification signal
DNA freeze–thaw stability	Bias of the cell number quantified in the frozen aliquot from that in the cooled aliquot within ± 40%	−14%–14%
Matrix effects in 14 mouse tissues	Tissue-specific recovery rates determined and matrix factors calculated	

Cal—calibration standard sample; Cq—quantification cycle; CV—coefficient of variation; LLOQ—lower limit of quantification; r^2^—correlation coefficient; QC—quality control standard sample; ULOQ—upper limit of quantification.

**Table 2:** Linear regression parameters of the calibration curves ^1^.

Assay No.	Objective	Slope	y-Intercept	Efficiency (%)	r^2^
1	Method validation	−4.8876	45.3621	60.177	0.990
2	Method validation, matrix effects	−3.9656	41.0232	78.718	0.971
3	Method validation, matrix effects	−3.5819	39.0348	90.187	0.992
4	Matrix effects	−3.6903	38.9021	86.630	0.993
5	Freeze–thaw stability	−4.6922	45.0217	63.350	0.994

^1^ Quantification range 125 to 20.000 human cells/200 µL lysate. r^2^—correlation coefficient.

**Table 3:** Calibration standard results.

Calibration Standard		Cal 1	Cal 2	Cal 3	Cal 4	Cal 5	Cal 6	Cal 7	Blank
Nominal cell number		20,000	10,000	5000	1000	500	250	125	0
Replicates per assay		3	3	3	3	3	6	6	6
Assay 1	Cq, mean	24.816	25.519	27.238	30.346	32.029	33.610	35.427 ^1^	36.233
	Cell number, mean (SD)	16,009 (1148)	11,478 (236)	5111 (248)	1189 (165)	535 (19)	258 (51)	110 (23) ^1^	74 (8)
	Bias (%)	−20	15	2	19	7	3	−12	-
	CV (%)	7	2	5	14	4	20	21	
Assay 2	Cq, mean	23.727	25.376	26.594	28.925	30.452	31.244	33.003 ^1^	38.867 ^2^
	Cell number, mean (SD)	23,121 (2970)	8961 (1921)	4486 (1407)	1162 (376)	485 (172)	314 (128)	109 (37) ^1^	3 ^2^
	Bias (%)	16	−10	−10	16	−3	25	−12	-
	CV (%)	13	21	31	32	36	41	34	
Assay 3	Cq, mean	23.311	24.711	26.149	28.362	29.592	30.298	31.498	39.735 ^3^
	Cell number, mean (SD)	24,567 (1347)	9994 (710)	3962 (202)	955 (47)	435 (53)	277 (40)	128 (19)	1 (1) ^3^
	Bias (%)	23	0	−21	−4	−13	11	3	-
	CV (%)	5	7	5	5	12	15	15	
Assays 1–3	n (total)	9	9	9	9	9	18	14	-
	Cell number, mean (SD)	21,232 (4581)	10,145 (1265)	4520 (575)	1102 (128)	485 (50)	283 (28)	116 (11)	
	Inter-assay bias (%)	6	1	−10	10	−3	13	−7	
	Inter-assay CV (%)	22	12	13	12	10	10	9	

^1^ Two of the six Cq values were excluded from calculation to improve curve fitting. ^2^ Signal was detectable only in one of six replicates. ^3^ Signal was only detectable in four out of six replicates. Cq—quantification cycle; CV—coefficient of variation; SD—standard deviation.

**Table 4:** Quality control standard results.

Quality Control Standard		QC 1	QC 2	QC 3	QC 4	QC 5	NTC
Nominal cell number		15,000	5000	1250	625	125	0
Replicates per assay		3	3	3	3	6	3
Assay 1	Mean Cq	25.419	27.444	[36.747] ^1^	33.265	34.826	36.511 ^3^
	Cell number, mean (SD)	12,079 (1288)	4647 (398)	[61 (22)] ^1^	400 ^2^	152 (56)	66 ^3^
	Bias (%)	−19	−7	[−95] ^1^	−36	22	-
	CV (%)	11	9	[37] ^1^	n.d. ^2^	36	
Assay 2	Mean Cq	23.852	25.846	28.558	29.877	32.739	39.978 ^5^
	Cell number, mean (SD)	18,410 (2984) ^4^	6780 (1139)	1417 (316)	649 (68)	125 (28)	2 ^5^
	Bias (%)	23	36	13	4	0	-
	CV (%)	16	17	22	11	22	
Assay 3	Mean Cq	24.133	26.096	28.349	29.419	31.536	40.861 ^6^
	Cell number, mean (SD)	14,481 (908)	4112 (437)	965 (90)	484 (26)	126 (24)	0 ^6^
	Bias (%)	−3	−18	−23	−23	1	-
	CV (%)	6	11	9	5	19	
Assays 1–3	n (total)	8	9	6	8	18	-
	Cell number, mean (SD)	14,990 (3196)	5180 (1412)	1191 (320)	511 (127)	135 (15)	
	Inter-assay bias (%)	0	4	−5	−18	8	
	Inter-assay CV (%)	21	27	27	25	11	

^1^ Value was excluded from further evaluation due to high bias of all three replicate measurements. ^2^ N = 1, two of the three cell number values were excluded from calculation due to high bias. ^3^ N = 2, one of the three replicates was excluded due to a pipetting error. ^4^ N = 2, one of the three values was excluded from calculation due to high bias. ^5^ Signal was only detectable in two out of three replicates. ^6^ Signal was only detectable in one out of three replicates. Cq—quantification cycle; CV—coefficient of variation; n.d.—not determined; NTC—no-template control; SD—standard deviation.

**Table 5:** Spike recovery in mouse tissues and blood spiked with human ABCB5^+^ MSCs ^1^.

Tissue ^2^	Spike Recovery Rates (%)									Matrix factor ^3,4^
	Assay 2 Mean (% CV)	Assay 3 Mean (% CV)	Assay 4 Mean (% CV)	Assays 2–4			Assays 2 and 3 ^3^			
				Mean	SD	% CV	Mean	SD	% CV	
Skin	144 (45)	147 (22)	29 (28)	107	67	63	146	2	1	0.68
Muscle	131 (29)	121 (5)	31 (38)	94	55	58	126	7	6	0.79
Lymph nodes	113 (38)	65 (27)	45 (16)	74	35	47	89	34	38	1.12
Liver	249 (49)	98 (7)	26 (42)	124	114	92	174	107	62	0.57
Spleen	37 (94)	45 (36)	12 (38)	31	17	55	41	6	14	2.44
Lung	28 (94)	48 (56)	10 (23)	29	19	66	38	14	37	2.63
Brain	19 (11)	48 (38)	11 (70)	26	19	75	34	21	61	2.94
Bone	38 (23)	81 (26)	13 (21)	44	34	78	60	30	51	1.67
Kidney	33 (25)	49 (29)	12 (10)	31	19	59	41	11	28	2.44
Thymus	70 (23)	70 (37)	21 (29)	54	28	53	70	0	0	1.43
Thyroid	100 (44)	62 (34)	42 (35)	68	29	43	81	27	33	1.23
Ovaries	49 (14)	28 (24)	23 (11)	33	14	41	39	15	39	2.56
Testes	18 (39)	39 (43)	17 (59)	25	12	50	29	15	52	3.45
Blood	10 (80)	11 (20)	3 (22)	8	4	54	11	1	7	9.09

^1^ Tissue homogenates/blood samples from SCID/beige mice were spiked with 5000, 625, 125, and 0 (blank samples) human skin-derived ABCB5^+^ MSCs per 200 µL lysate. Three independent assays (assays 2, 3, and 4) were performed on three different days. Within each assay, samples spiked with 5000 and 625 cells were assayed in triplicate, and samples spiked with 125 cells and blank samples in sextuplicate. ^2^ For tissue concentrations, see Table S1. ^3^ Since the spike recovery rates for almost all the tissues were considerably lower in assay 4 as compared to assays 2 and 3, possibly indicating inefficient DNA extraction, the data were re-analyzed for assays 2 and 3 alone. ^4^ Matrix factor = 100/mean recovery rate. CV—coefficient of variation; MSC—mesenchymal stromal cell; SD—standard deviation.

**Table 6:** Tissue and blood components that reported to negatively affect qPCR.

Inhibitor	Tissue	Mode of Action	References
Melanin	Skin, melanoma metastases	Reversible binding to thermostable DNA polymerases	[44]
		Binding to DNA, thereby limiting the amount of available template	[45]
Myoglobin	Muscle	Inhibition of *Taq* DNA polymerase	[46]
Collagen	Bone	Inhibition of thermostable DNA polymerases, binding to template DNA	[45,47]
Calcium ions		Inhibition of thermostable DNA polymerases, likely through competition with the polymerase cofactor Mg^2+^	[45,47]
Hemoglobin	Blood	Impairment of DNA polymerase activity, fluorescence quenching through binding to or interacting with fluorescent dyes	[48]
Immunoglobulin G		Binding to single-stranded genomic DNA, thereby hindering the primer annealing or binding of DNA polymerase	[48]
Lactoferrin		Release of iron ions	[49]
EDTA ^1^		Chelation of the polymerase cofactor Mg^2+^	[50,51]
Heparin ^1^		Competition with template DNA, chelation of the polymerase cofactor Mg^2+^	[49,51]

^1^ Used as anticoagulant.

## Data Availability

The data presented in this study are available on request from the corresponding author.

## Supplementary Materials

The following supporting information can be downloaded at: https://www.mdpi.com/article/10.3390/cells12131788/s1, Table S1: Validated tissue concentrations and homogenization cycle numbers for preparation of tissue quality control standards; Table S2: Tissue quality control sample results; Table S3: Freeze–thaw stability of extracted DNA isolates from the quality control standards; Table S4: Spike recovery rates with different DNA extraction protocols from mouse blood samples.
