# Supplementary material for: Drug Regulatory-Compliant Validation of a qPCR Assay for Bioanalysis Studies of a Cell Therapy Product with a Special Focus on Matrix Interferences in a Wide Range of Organ Tissues
Source: Cells. 2023 Jul 5;12(13):1788. doi: 10.3390/cells12131788 (PMC10340683; doi:10.3390/cells12131788)
Supplement: Supplementary file 1 [file cells-12-01788-s001.zip › cells-2443177-supplementary.pdf]

Table S1. Validated tissue concentrations and homogenization cycle numbers for preparation of tissue quality control standards using various tissues from SCID/beige mice

**Tissue**	**Tissue concentration [mg/ml] [1]**	**Number of homogenization cycles**	**Bead type**
Skin	100	7	Ceramic
Muscle	100	7	Ceramic
Lymph nodes [2]	50	4	Ceramic
Liver	100	3	Ceramic
Spleen	100	7	Ceramic
Lung	100	6	Ceramic
Brain	100	3	Ceramic
Bone	100	2+2 [3]	Steel
Kidney	100	4	Ceramic
Thymus	100	4	Ceramic
Thyroid	30	5	Ceramic
Ovaries	80	4	Ceramic
Testes	100	3	Ceramic

[1] Concentrations were adapted with lysis buffer T1 (NucleoSpin® 96 Tissue kit, Macherey-Nagel).

[2] Cervical, axial and inguinal lymph nodes pooled.

[3] Two cycles without lysis buffer T1 (dry) and two cycles after lysis buffer T1 addition.

Table S2. Tissue quality control sample results [1]

Sample		TQC1		TQC2		TQC3		TBLK		
Nominal cell count [2]		5000		625		125		0		
Replicates per assay		3		3		6		6		
Tissue		Cq	Calculated cell number	Cq	Calculated cell number	Cq	Calculated cell number	Number of replicates with detected signal	Cq	Calculated cell number
**Skin**	Assay 2	26.423	4836	29.726	725	31.421	273	5	37.112	11
	Assay 3	25.020	8200	28.898	681	30.732	210	6	39.294	1
	Assay 4	26.804	1901	30.832	154	33.522	29	5	39.316	1
**Muscle**	Assay 2	26.586	4412	29.134	1005	32.092	182	6 [3]	34.192	57 [3]
	Assay 3	25.576 [4]	5721 [4]	28.687	797	31.251	150	6	37.780	3
	Assay 4	26.840	1857	31.407	108	32.738	48	6	36.331	6
**Lymph nodes**	Assay 2	26.721	4052	30.003	605	31.886	203	5	37.697	9
	Assay 3	26.144	4011	30.234	287	32.157	85	6	38.489	2
	Assay 4	26.503	2296	30.153	236	32.218	65	5	38.296	2
**Liver**	Assay 2	25.577	7954	28.745	1255	33.387	485	5	37.089	10
	Assay 3	25.921	4587	29.080	602	32.595	131 [5]	6	32.980 [6]	49 [6]
	Assay 4	27.062	1617	30.363	208	34.428	17	1	37.187	3

	Sample	TQC1		TQC2		TQC3		TBLK		
	Nominal cell count [2]	5000		625		125		0		
	Replicates per assay	3		3		6		6		
**Tissue**		Cq	Calculated cell number	Cq	Calculated cell number	Cq	Calculated cell number	Number of replicates with detected signal	Cq	Calculated cell number
**Spleen**	Assay 2	29.000	1138	33.247	72 [3]	33.677	96 [3]	3	38.797	4
	Assay 3	26.850	2594	31.087	168	32.428	72	3	40.092	1
	Assay 4	28.007	897	32.795	62	35.347	12	2	39.477	1
**Lung**	Assay 2	29.842	668	32.928	78 [3]	34.011	73 [3]	3	39.296	3
	Assay 3	27.006	1851 [3]	30.975	179	31.940	99	4	39.009	1
	Assay 4	29.402	377	32.348	61	34.860	15	2	38.864	1
**Brain**	Assay 2	29.464	836	32.253	129 [3]	35.731	23	3	37.121	26
	Assay 3	26.791	3376 [3]	30.509	193 [3]	32.739	58	4	38.664	1
	Assay 4	28.768	558	34.194	20	33.971	23	6	39.083	1
**Bone**	Assay 2	28.576	1399	31.490	256	34.313	55	4	40.446	2
	Assay 3	26.203	3840	29.369	388 [3]	31.490	129	2	38.553	1
	Assay 4	28.241	778	32.026	74	35.360	13	1	39.683	1
**Kidney**	Assay 2	28.613	1365	32.324	184	34.503	53	3	38.596	5
	Assay 3	26.647	3118	30.704	213	32.647	63	2	40.851	0
	Assay 4	28.753	563	32.193	67	34.629	16	0	n.d.	n.d.

Tissue	Sample / Nominal cell count [2] / Replicates per assay	TQC1 Cq	TQC1 Calculated cell number	TQC2 Cq	TQC2 Calculated cell number	TQC3 Cq	TQC3 Calculated cell number	TBLK Number of replicates with detected signal	TBLK Cq	TBLK Calculated cell number
	**Sample**	**TQC1**		**TQC2**		**TQC3**		**TBLK**		
	Nominal cell count [2]	5000		625		125		0		
	Replicates per assay	3		3		6		6		
**Thymus**	Assay 2	27.522	2597	30.433	470	33.064	104	5	38.495	5
	Assay 3	27.189	2044	29.651	508	31.923	111	2	39.574	1
	Assay 4	27.318	1378	31.508	102	33.945	23	4	39.265	1
**Thyroid**	Assay 2	27.240	3001	29.283	919	32.869	116	4	35.945	27
	Assay 3	26.771	2669	29.277	536	32.751	58	6	37.907	2
	Assay 4	27.106	1574	29.424	370	32.817	46	5	38.053	2
**Ovaries**	Assay 2	27.909	2047	31.096	320	33.931	68	2	39.598	2
	Assay 3	27.746	1426	31.488	129	33.244	43	3	38.530	2
	Assay 4	27.667	1109	31.117	129	33.429	32	2	41.588	0
**Testes**	Assay 2	30.175	548	32.826	120	35.161	32	2	39.271	3
	Assay 3	27.522	1639	31.142 [3]	165 [3]	32.427	72	2	40.326	0
	Assay 4	27.263	1434	31.781	87	35.010	12 [3]	2	39.042	1
**Blood**	Assay 2	31.295	287	35.459	31 [3]	36.091	24	2	38.693	4
	Assay 3	29.163	571	32.962	52	34.809	16	3	40.138	1
	Assay 4	30.656	172	34.678	15	36.714	5	1	36.569	4

[1] Tissue homogenates/blood from SCID/beige mice were spiked with human skin-derived $ABCB5^+$ mesenchymal stromal cells. Data are means of 3 or 6 replicates.

[2] In 200 µl lysate.

[3] One value was excluded due to high CV.

[4] One replicate was not determined and another replicate was excluded due to high CV.

[5] Calculated cell numbers were multiplied by 2, because TQC2 was accidently mixed with TBLK before DNA extraction.

[6] Values represent accidental mixture of BLK with TQC2.

Cq – quantification cycle; CV – coefficient of variation; n.d. – not detectable.

Table S3. Freeze-thaw stability of extracted DNA isolates from the quality control standards

**Quality control standard**		**QC1**	**QC2**	**QC3**	**QC4**	**QC5**
Nominal cell number (in 200 µl lysate)		15,000	5000	1250	625	125
Number of replicates per assay		3	3	3	3	6
**Storage temperature 2 to 8 °C**	Mean Cq	24.529	26.829	29.144	30.255	32.737
	Mean cell number (SD)	23,338 (1568)	7604 (1220)	2426 (217)	1425 (296)	419 (49)
	Precision, % CV	7	16	9	21	12
**Storage temperature -25 to -15 °C**	Mean Cq	24.294	26.879	29.454	29.959	32.809
	Mean cell number (SD)	26,195 (1938)	7411 (1155)	2095 (312)	1626 (139)	411 (99)
	Precision, % CV	7	16	15	9	24
**% Bias (frozen vs. cooled aliquot)** [1]		12	-3	-14	14	-2

[1] Percent bias of mean cell number in the frozen aliquot from mean cell number in the cooled aliquot.

Cq – quantification cycle; CV – coefficient of variation; SD – standard deviation.

Table S4. Spike recovery rates with different DNA extraction protocols from mouse blood samples spiked with human ABCB5^{+} MSCs

**DNA extraction protocol**	**A**			**B**	**C**		**D**	**E**
Blood	100 µl			100 µl	100 µl		50 µl	100 µl
PBS	ad 400 µl			ad 400 µl	80 µl		ad 200 µl	ad 200 µl
Proteinase K	25 µl			50 µl	25 µl		25 µl	25 µl
Binding buffer BQ1	400 µl			400 µl	200 µl		200 µl	200 µl
Incubation	RT 5 min,<br>70 °C 15 min			RT 30 min,<br>70 °C 15 min	RT 0 min<br>70 °C 15 min		RT 5 min,<br>70 °C 15 min	RT 5 min,<br>70 °C 15 min
% Spike recovery (% CV)	10 (80)	11 (20)	9 (20)	3 (22)	9 (22)	4 (48)	8 (13)	4 (31)

Changes from protocol A are highlighted in yellow. CV – coefficient of variation; MSC – mesenchymal stromal cell; PBS – phosphate-buffered saline.
